# Endothelin-1–Endothelin receptor B complex contributes to oligodendrocyte differentiation and myelin deficits during preterm white matter injury

**DOI:** 10.3389/fcell.2023.1163400

**Published:** 2023-03-17

**Authors:** Mengjie Du, Na Wang, Xiaolong Xin, Chun-Lan Yan, Yan Gu, Liang Wang, Ying Shen

**Affiliations:** ^1^ Department of Pathology, The Second Affiliated Hospital, Zhejiang University School of Medicine, Hangzhou, China; ^2^ Department of Physiology and Department of Psychiatry, Sir Run Run Shaw Hospital, Zhejiang University School of Medicine, Hangzhou, China; ^3^ NHC and CAMS Key Laboratory of Medical Neurobiology, Institute of Neuroscience and Department of Neurology of the Second Affiliated Hospital, Zhejiang University School of Medicine, Hangzhou, China; ^4^ Department of Stem Cell Biology, Zhejiang University School of Medicine, Hangzhou, China

**Keywords:** white matter injury, neuron-glia interaction, oligodendrocyte precursor cell, single-cell RNA sequencing, myelination

## Abstract

Preterm cerebral white matter injury (WMI), a major form of prenatal brain injury, may potentially be treated by oligodendrocyte (OL) precursor cell (OPC) transplantation. However, the defective differentiation of OPCs during WMI seriously hampers the clinical application of OPC transplantation. Thus, improving the ability of transplanted OPCs to differentiate is critical to OPC transplantation therapy for WMI. We established a hypoxia–ischemia-induced preterm WMI model in mice and screened the molecules affected by WMI using single-cell RNA sequencing. We revealed that endothelin (ET)-1 and endothelin receptor B (ETB) are a pair of signaling molecules responsible for the interaction between neurons and OPCs and that preterm WMI led to an increase in the number of ETB-positive OPCs and premyelinating OLs. Furthermore, the maturation of OLs was reduced by knocking out ETB but promoted by stimulating ET-1/ETB signaling. Our research reveals a new signaling module for neuron–OPC interaction and provides new insight for therapy targeting preterm WMI.

## Introduction

Cerebral white matter injury (WMI) is a major form of brain injury that occurs in preterm infants, resulting in adolescent neurological disability, such as spastic motor deficits (cerebral palsy) and cognitive impairment (Volpe, 2009). Based on the pathological characteristics, preterm WMI is classified into diffuse WMI, brain-penetrating cyst, periventricular leukomalacia (PVL), or focal WMI (Buser et al., 2012; van Tilborg et al., 2016). Although the pathogenesis of WMI is unclear, it can be caused by ischemia and hypoxia, infection/inflammation, or excitotoxicity, which reduces white matter volume and white matter bundle and subsequently leads to myelination deficiency and axon encapsulation in the central nervous system (CNS) (Volpe, 2001; Blumenthal, 2004; Alexandrou et al., 2014). Thus, stimulating myelin formation is an effective strategy for the clinical treatment of infants suffering from preterm WMI.

The main cell type affected in preterm WMI is the oligodendrocyte precursor cell (OPC). The loss or arrested differentiation of OPCs is the main cause of hypomyelination and the reduced volume of white matter following preterm WMI (Motavaf and Piao, 2021). Thus, restoring the normal development of OPCs is crucial for the treatment of preterm WMI. In line with this speculation, OPC transplantation in animals has been shown to effectively increase recovery from preterm WMI and spinal cord injury (Porambo et al., 2015). Nevertheless, the differentiation of transplanted OPCs is severely inhibited by the adverse microenvironment caused by brain injury, albeit they can migrate to the lesions (Tepavcevic and Lubetzki, 2022). Given that improving the capacity of transplanted OPCs to differentiate is a key step for the treatment of preterm WMI, uncovering the molecular mechanisms controlling OPC differentiation during preterm WMI is essential to OPC transplantation therapy.

Multiple intrinsic and extrinsic molecules are known to play roles in the differentiation of OPCs. On one hand, a variety of OPC-derived regulatory factors promote their differentiation. For example, transcriptional regulators, such as *Olig1*, *Olig2*, *MRF*, *Sox10*, and *Nkx2.2*, promote the development of OPCs, whereas other transcriptional factors, such as *Sox2* and *Sox6*, inhibit their differentiation (Emery, 2010; Adams et al., 2021; Clayton and Tesar, 2021). On the other hand, neuron-secreted molecules such as Jagged, PAS-NCAM, and Lingo-1 also regulate the differentiation of OPCs by binding to their corresponding receptors on OPCs (Wang et al., 1998). However, the expression profile of neuron-derived regulatory factors and their effects on OPC differentiation during WMI are still elusive.

In the present work, we established a hypoxia–ischemia-induced preterm WMI model in mice and screened OPCs using single-cell RNA sequencing (scRNA-seq) to identify neuron-derived molecules affected by preterm WMI. We found that endothelin (ET)-1 and endothelin receptor B (ETB) were mainly expressed in neurons and OPCs, respectively; the number of ETB^+^ OPCs was significantly increased after preterm WMI; and the maturation of oligodendrocytes (OLs) was inhibited by the ablation of ETB, but promoted by stimulating ET-1.

## Materials and methods

### Mice

All animal experiments were approved and performed according to the guidelines of the Institutional Animal Care and Use Committee of Zhejiang University. Mice were housed in a temperature- and humidity-controlled facility under a 12:12-h light–dark cycle. *Ednrb* ^+/−^ (#003295; RRID:IMSR_JAX:003,295) mice were purchased from the Jackson Laboratory (Bar Harbor, ME).

### Antibodies and reagents

Antibodies against the following were used: Olig2 (#ab9610; RRID:AB_570666), SOX10 (#AB5727; RRID: AB_2195375), MBP (#SMI99; RRID: AB_2314772), NeuN (#MAB377; RRID: AB_2298772), PH3 (#05-806; RRID: AB_310016), and GAPDH (#MAB374, RRID: AB_2107445) from Millipore (Billerica, MA); GFAP (#Z0334; RRID: AB_10013382) from Dako (Copenhagen, Denmark); Iba1 (#019-19741; RRID: AB_839504) from Wako (Tokyo, Japan); caspase-3 (#9661; RRID: AB_2341188) from Cell signaling (Boston, MA); and A2B5 (#MAB312; RRID: AB_94709), O4 (#MAB345; RRID: AB_11213138), and O1 (#MAB344; RRID: AB_94860) from Sigma-Aldrich (St. Louis, MO). Alexa-405, Alexa-488, Alexa-555, and Alexa-637 secondary antibodies were obtained from Life Technologies. Dulbecco’s modified Eagle’s medium, 4′,6-diamidino-2-phenylindole, Neurobasal, and B27 supplements were obtained from Invitrogen (Carlsbad, CA). Triiodothyronine (#T6397) and other chemicals were obtained from Sigma-Aldrich unless stated otherwise.

### Neonatal mouse WMI model

P3 mice were placed in a refrigerator at −20°C for 7–10 min, and immediately the right common carotid artery was carefully ligated. The wound was closed with an 8–0 suture. The time of operation was controlled to within 5 min. After surgery, the pups were allowed to recover under a heat lamp for 10 min, returned to their mother, and again allowed to recover for 1 h. Next, the pups were exposed to 6% oxygen (94% nitrogen saturation) at 37°C for 90 min in a humidified chamber and returned to their cages with external monitoring.

### scRNA-seq

Control and WMI brains were dissected in Leibovitz’s L-15 medium (Life Technologies), and the tissue was incubated in 0.05% trypsin–EDTA (Life Technologies) for 15 min in a thermomixer comfort (Eppendorf) at 700 rpm. Following enzymatic digestion, the tissue was triturated using two pipettes of different sizes for homogenization. The cell suspension was filtered through a 70-μm cell strainer (BD Biosciences) to remove clusters and then subjected to scRNAseq analysis (Lianchuan Biotech, Hangzhou). The cells from either control or WMI brain (four replicates for each) were integrated using Seurat’s IntegrateData function. Feature selection was applied with Seurat using default parameters. A feature of individual cells (gene expression, PC score, or the number of genes detected) was colored for UMAP dimensional reduction plots, given Seurat R-object (Robj) after the clustering step. RNA-seq data have been uploaded to the repository with the URL: https://www.ncbi.nlm.nih.gov/geo/query/acc.cgi?acc=GSE225580.

### Immunohistochemistry and immunocytochemistry

Mouse brains were perfused with phosphate-buttered saline (PBS) followed by 4% paraformaldehyde (PFA) and fixed in PFA at 4°C overnight. After immersion in 30% sucrose in PBS, the brains were embedded in OCT and cut coronally into 60-μm sections on a cryostat (Leica CM 1860). The sections were incubated with primary antibodies overnight at room temperature (RT), washed with PBS, and incubated with secondary antibodies for 1 h at RT. Cultured cells were fixed in 4% paraformaldehyde and 4% sucrose for 15 min at RT, washed with PBS, permeabilized in 0.2% Triton X-100 for 10 min, blocked in 10% bovine serum albumin for 1 h, and labeled with primary antibodies overnight at 4°C. Nuclei were counterstained with 4′6-diamidino-2-phenylindole (Vector Laboratories). The dilution ratios were 1:100 (Olig2), 1:250 (MBP), 1:500 (GFAP), and 1:1,000 (SOX10, NeuN, Iba1, caspase-3, PH3, Alexa-405, Alexa-488, Alexa-555, and Alexa-637).

### RNAscope

Brains were cut into 20-μm sections on a cryostat (Leica). Fluorescence *in situ* hybridization was applied using an advanced cell diagnostics RNAScope Multiplex Fluorescent Kit (ACD: #320850; Newark, CA). The *Ednrb* probe (#473801; targeting 473–1,481 bp of NM_007904.4) and the *Edn1* probe (#435221; targeting 902–2053 bp of NM_010104.3) were manufactured by Advanced Cell Diagnostics. After hybridization, the sections were processed for immunostaining.

### Microscopy and imaging

A FLUOVIEW FV3000 confocal microscope (Olympus, Tokyo, Japan) was used to capture fluorescent images, which were further processed using Photoshop CS 8.0 (Adobe, San Jose, CA; RRID:SCR_014199). The parameters used in microscopy were consistent in all experiments. ImageJ 1.42q (NIH, Bethesda, MD; RRID:SCR_003070) was used to count cells and analyze fluorescence intensity, as in previous research (Ma et al., 2019). The images of at least nine representative fields were acquired from each section. To count the number of cells in one experiment, two sections from one mouse and at least four mice from each group were collected.

### OPC culture and OGD

OPCs from newborn (P0) Sprague–Dawley rats were cultured based on previous research (Zhou et al., 2014; Xie et al., 2018). OPCs were collected from mixed cortical glial cultures by shaking for 1 h at 200 rpm, incubated in fresh DMEM for 4 h, and shaken at 250 rpm at 37 °C for 16 h. Collected OPCs were replated onto poly-D-Lysine-coated plates and grown in Neurobasal supplemented with 2% B27. The purity of OPC culture was usually >90%. After harvesting, OPCs were exposed to PDGF-AA (10 ng/mL) for 3 days to keep them undifferentiated or to T3 (40 ng/mL) for 3 days to stimulate differentiation. IgG-Fc (100 ng/mL in PBS; #1460-CD-050; R&D Systems) or ET-1 (100 ng/mL in PBS; #1160; R&D Systems) was applied 10 min before OGD. Cell death was quantitatively assessed by using a trypan blue exclusion method.

### Western blotting

Proteins were rinsed with PBS and diluted in 1% SDS containing a protease inhibitor cocktail. Protein concentration was determined by the BCA assay. Equal quantities of protein were loaded onto sodium dodecyl sulfate–polyacrylamide gel, transferred to PVDF membranes (Immobilon-P, Millipore), immunoblotted with antibodies, and visualized by enhanced chemiluminescence (Pierce Biotechnology, Rockford, IL). The primary antibody dilution ratios were 1:10,000 for MBP and 1:20,000 for GAPDH. Film signals were digitally scanned and quantified using ImageJ.

### Statistics

Data statistics and illustrations were generated using SPSS 16.0 (SPSS), GraphPad Prism 7.0 (GraphPad Software), and CorelDRAW 12.0 (Corel Corp.). Statistically significant differences were determined using unpaired two-sided Student’s t-test for two-group comparison or one-way ANOVA followed by the LSD *post hoc* test for multiple comparisons. The accepted level of significance was *p* < 0.05. “*n*” represents the number of animals or cultures. All data in the text and figures are presented as the mean ± SEM.

## Results

### Preterm WMI injury does not affect the survival or proliferation of OPCs

**FIGURE 1:**
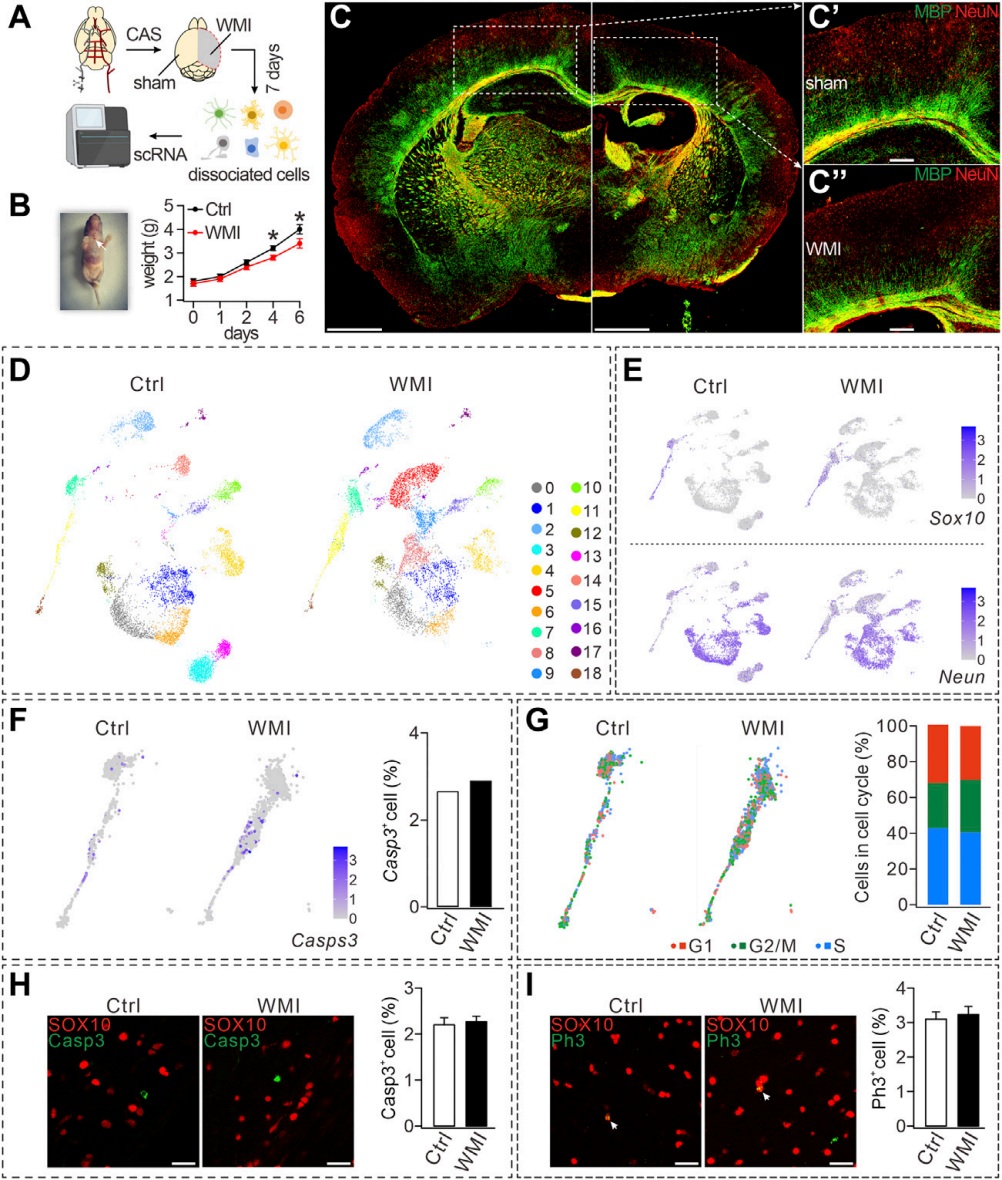
CAS-induced WMI and scRNA-Seq analysis. **(A)** Schematic diagram of scRNA-Seq of sham and WMI cortex. P3 mice were euthanized on day 7 after unilateral CAS. Sham and WMI hemispheres were dissected and dissociated into a single-cell suspension. Single cells and barcoded beads were captured into droplets for RNA-Seq. **(B)** Left: image of a mouse after the surgery and wound ligation (arrowhead); Right: body weight after CAS surgery in control (Ctrl) and WMI mice. Day 4: 3.2 ± 0.1 g (Ctrl; *n* = 9) and 2.8 ± 0.1 g (WMI; *n* = 9), *p* = 0.030. Day 6: 4.0 ± 0.2 g (Ctrl; *n* = 9) and 3.4 ± 0.2 g (WMI; *n* = 9), *p* = 0.044. **(C)** Coronal view of a mouse brain (P10) subjected to unilateral CAS with MBP and NeuN staining. The averages of MBP fluorescent intensity were 3,311 ± 507 (Ctrl; *n* = 4) and 1,167 ± 142 (WMI; *n* = 4), *p* = 0.015. **(C’)** and **(C”)** are magnifications of the boxed regions in **(C)**. Scale bars: 1 mm (whole brain); 250 μm (magnifications). **(D)** UMAP graph showing unsupervised clustering of cells (P10 cerebral cortex). Distinct cell types are highlighted by different colors. **(E)** Gene expression profiles of *Sox10* and *Neun* visualized *via* UMAP. The mRNA level is shown on a linear scale. Percentages of *Sox10* ^*+*^ cells: 8.8% (control; *n* = 4) and 10.0% (WMI; *n* = 4). Percentages of *Neun* ^*+*^ cells: 74.5% (control; *n* = 4) and 70.5% (WMI; *n* = 4). **(F)** Gene expression of caspase-3 (*Casps-3*) in an OL cluster. Percentages of caspase-3^*+*^ cells: 2.7% (control; *n* = 4) and 2.9% (WMI; *n* = 4). **(G)** *Sox10*-marked OLs partitioned into three clusters expressing the G1/S program, G2/M program, or non-cycling. In control: 43.0% (G1), 25.1% (G2/M), and 31.9% (S). In WMI: 40.5% (G1), 29.4% (G2/M), and 30.1% (S). **(H)** Expression of SOX10 and caspase-3 in P10 cerebral cortex: 2.2% ± 0.3% (control; *n* = 4) and 2.3% ± 0.8% (WMI; *n* = 4), *p* = 0.42. Scale bars: 50 μm. **(I)** Expression of SOX10 and PH3 in P10 cerebral cortex: 3.1% ± 0.3% (control; *n* = 4) and 3.2% ± 0.8% (WMI; *n* = 4), *p* = 0.67. Scale bars: 50 μm *p < 0.05.

First, we established a WMI model, which was modified from a previous study (Wang et al., 2020), using carotid artery stenting (CAS) surgery on one hemisphere of the brain in postnatal day 3 (P3) mice (Figure 1A). In line with Wang et al. (2020), the body weight of WMI mice was significantly lesser than that of the control mice four days after the operation (Figure 1B). To evaluate the pathological characteristics of WMI, we applied immunostaining for MBP (myelin basic protein), a myelin marker protein, to the mice 7 days after CAS surgery. We found that white matter tracts decreased and the corpus callosum became thinner on the surgical (WMI) side of the brain, indicating that the WMI induced myelin dysplasia (Figure 1C). In contrast, no myelin defect was found on the unaffected (control) side (Figure 1C). Therefore, P10 mice (7 days after surgery) were chosen to investigate the effects of WMI.

To evaluate cell-type-specific transcription, scRNA-seq was applied to cells dissociated from the cortex on both the control and WMI sides (*n* = 4), and then a total of 14,082 single cells (7,307 from the control and 6,775 from the WMI hemisphere) were sequenced. The harvested cells showed significant cellular heterogeneity: 19 clusters in both the WMI and control groups with unsupervised Seurat clustering by uniform manifold approximation and projection (UMAP) (Figure 1D). Notably, cell numbers were similar in some clusters from both groups (clusters #0 and #1), but decreased in others (clusters #3 and #13) (Figure 1D), indicating that WMI surgery differentially affects cell types in the brain.

Based on the enriched expression of the neuronal marker *Neun*, clusters #1, #4, #12, and #14 were grouped and defined as neurons. In addition, based on the enriched expression of the OL marker *Sox10*, clusters #3, #11, and #18 were grouped and defined as OLs. Despite that neuron and OL clusters demonstrated distinct expression paradigms (Figure 1E), the numbers of both *Sox10* ^+^ and *Neun* ^+^ cells in the WMI group were comparable to those in the control group (Figure 1E), suggesting that neither neuron nor OL survival was affected by WMI. Furthermore, quantification of the expression of caspase-3 in OL clusters showed that, compared to controls, the number of caspase-3^+^ cells in OL clusters was not changed by WMI (Figure 1F), further supporting that WMI does not affect the survival of OLs. To reveal the cell-cycle states of OL clusters, we scored each cell for its cycle phase using the signatures of G1/S (DNA replication genes), G2/M (mitosis genes), and non-cycling (quiescent cells) phases. Our results demonstrated that the frequency of cells in G1/S and G2/M clusters (cycling cells) did not differ between control and WMI groups (Figure 1G), implying that WMI does not affect the proliferation of OPCs. These results from the scRNA-Seq analysis were further confirmed by the immune detection of caspase-3 and SOX10. We found that the percentage of caspase-3^+^ cells among SOX10^+^ cells did not differ between the control and WMI groups (Figure 1H). Similarly, double staining with the antibodies against PH3, a biomarker for the proliferation cycle, and SOX10 showed that the proliferative capacity of OPCs was also unaltered (Figure 1I). Collectively, our results showed that neither the survival nor the proliferation of OPCs is affected by WMI.

### OPC differentiation is arrested by WMI

**FIGURE 2:**
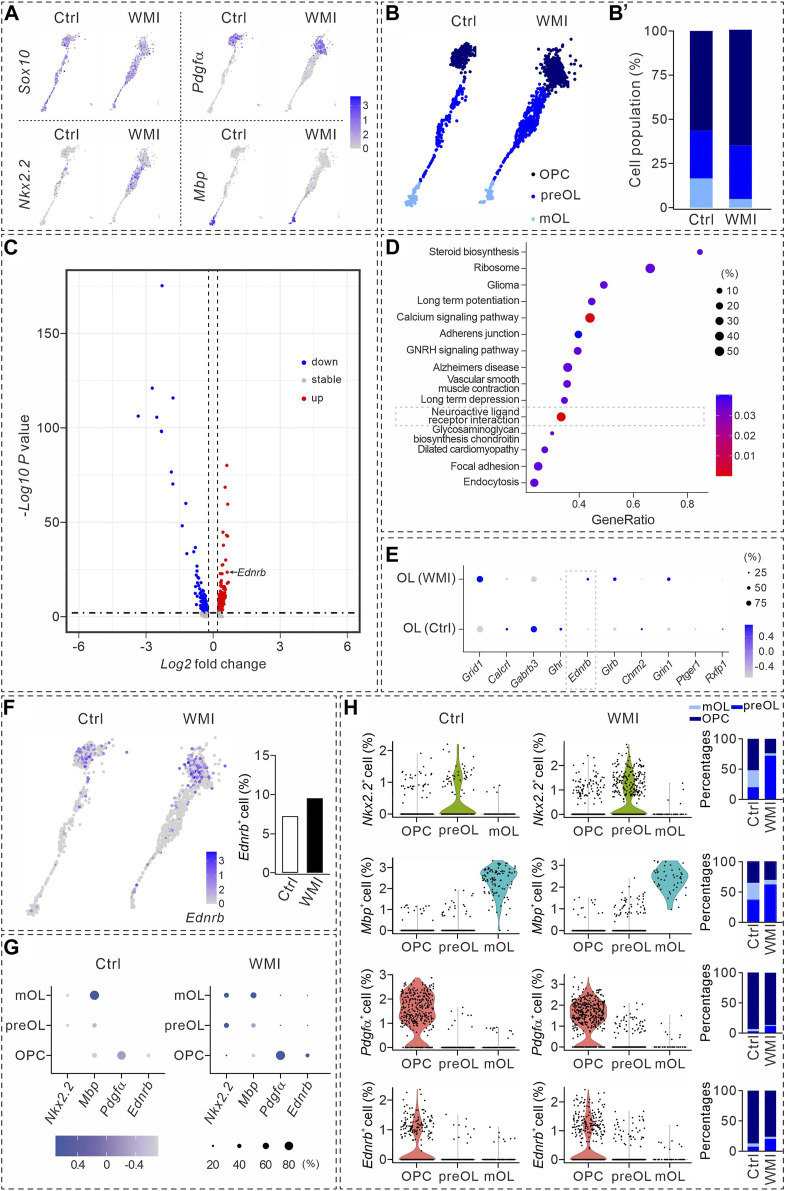
Transcriptional analysis during oligodendrocyte maturation. **(A)** UMAP plots showing the expression of *Sox10*, *Nkx2.2*, *Pdgfα*, and *Mbp* in OL clusters from P10 cerebral cortex. Averages in control: 57.4% (*Nkx2.2*), 57.1% (*Pdgfα*), and 13.5% (*Mbp*). Averages in WMI: 55.4% (*Nkx2.2*), 63.4% (*Pdgfα*), and 5.3% (*Mbp*). **(B)** *Sox10* ^*+*^ cells partitioned into OPC, pre-OL, and mature OL (mOL) clusters. **(B’)** Averages in control: 56.4% (OPC), 27.1% (pre-OL), and 16.5% (mature OL). Averages in WMI: 64.8% (OPC), 30.4% (pre-OL), and 4.9% (mature OL). **(C)** Volcano plot derived from the single-cell transcriptomic analysis showing the differential gene expression pattern of OLs in control and WMI OLs. *Y*-axis: level of significance of each gene; *X*-axis: difference in the expression level of each gene. **(D)** Top 15 KEGG pathways revealed by GO analysis (dashed box: activated neuroactive ligand and receptor interaction pathways). **(E)** Dot plot showing differentially regulated genes in neuroactive ligand and receptor interaction pathways. Note the upregulation of *Ednrb* in the WMI group. Color scale: z-score; dot size: percentage. **(F)** UMAP plots show the expression of *Ednrb* in OL clusters from the control and WMI cerebral cortex (P10). **(G)** Marker gene expression across the three OL subpopulations. Dot size is proportional to the percentage of marker genes of each cluster, and blue color intensity is correlated with the expression level. **(H)** Violin plots of *Ednrb*, *Nkx2.2*, *Pdgfα*, and *Mbp* from the clusters defined in **(G)**. *Nkx2.2*: For control, 52% (OPC), 20% (preOL) and 28% (mOL); For WMI, 24% (OPC), 72% (preOL) and 4% (mOL). *Mbp*: for control, 36% (OPC), 37% (preOL) and 28% (mOL); for WMI, 31% (OPC), 62% (preOL), and 7% (mOL). *Pdgfα*: for control, 93% (OPC), 3% (preOL), and 4% (mOL); for WMI, 87% (OPC), 12% (preOL), and 1% (mOL). *Ednrb*: for control, 87% (OPC), 7% (preOL), and 6% (mOL); for WMI, 76% (OPC), 20% (preOL), and 4% (mOL).

We next analyzed the cellular composition of the OL clusters, within which three types of *Sox10* ^+^ cells were identified (*Pdgfα* ^+^ OPCs, *Nkx2.2* ^+^ premyelinating OLs or pre-OLs, and *MBP* ^+^ mature OLs). Notably, the numbers of OPCs and pre-OLs within the WMI clusters were higher than those in the control clusters (Figure 2A). Together with the reduced number of *Mbp* ^+^ OLs in the WMI group (Figure 2A), we speculated that WMI might inhibit the differentiation of OPCs, thereby arresting OLs at the OPC stage. To test this point, three types of OLs were quantified in the control and WMI groups. Indeed, our results demonstrated that the percentage of mature OLs in the WMI group was reduced, while the percentages of both OPCs and pre-OLs in the WMI group were increased (Figures 2B, B’). Thus, we concluded that most of the OLs were restricted to immature stages following WMI.

### Specific expression of *Ednrb* in OPCs and its regulation by WMI

To identify the molecules involved in the inhibition of OPC differentiation after WMI, we compared the transcriptomes of OPCs and pre-OLs derived from the control and WMI scRNA-seq datasets. Our results showed that ∼200 genes were upregulated, 400 were downregulated, and 20 genes remained stable when OLs were challenged by WMI (Figure 2C). The characterization of differentially expressed genes by a DAVID cellular component Gene Ontology analysis revealed that the neuroactive ligand receptor interaction pathway was one of the most abundant functional classes with WMI (Figure 2D). Among the changed genes in this interaction pathway, we noted that the expression of a previously unrecognized gene, *Ednrb*, was significantly increased in the population of WMI OLs (Figure 2E), suggesting that *Ednrb* might be a candidate gene involved in defective OPC differentiation during WMI. Indeed, *Ednrb* was expressed in the OL clusters of both the control and WMI groups and the number of *Ednrb* ^+^ cells increased following WMI (Figure 2F).

We validated the expression of *Ednrb* in subpopulations of OLs. As expected, OPCs, pre-OLs, and mature OLs displayed enriched expression of *Nkx2.2*, *Mbp*, and *Pdgfrα* genes, respectively (Figure 2G). In addition, we noticed that *Pdgfrα*+ was relatively higher expressed in WMI OPCs, and both the expression intensity of *Ednrb* and the percentage of *Ednrb* ^+^ cells in the WMI cluster were slightly higher than those in the control cluster (Figure 2G). Meanwhile, the expression intensity of *Nkx2.2*, *Mbp, Pdgfrα*, or *Ednrb* of each subpopulation was also analyzed using violin plots (Figure 2H), which indicated that *Ednrb* is tightly associated with OPCs and increases after WMI.

### ET-1/ETB is a signaling complex for neuron–OPC interaction

The specific expression of *Ednrb* in immature OLs suggests that it plays unique roles during OL differentiation. It has been established that *Ednrb* encodes endothelin receptor B (ETB), a member of the G-protein-coupled receptor family, and is a receptor of ET-1 (Welch et al., 2013). Previous studies have shown that ETB is expressed in cancer cells and renal collecting ducts and interacts with ET-1 to impact benign and malignant tissues through vasoconstriction (Grimshaw, 2007). However, relatively little is known about the function of the ET-1/ETB complex in the central nervous system (CNS).

**FIGURE 3:**
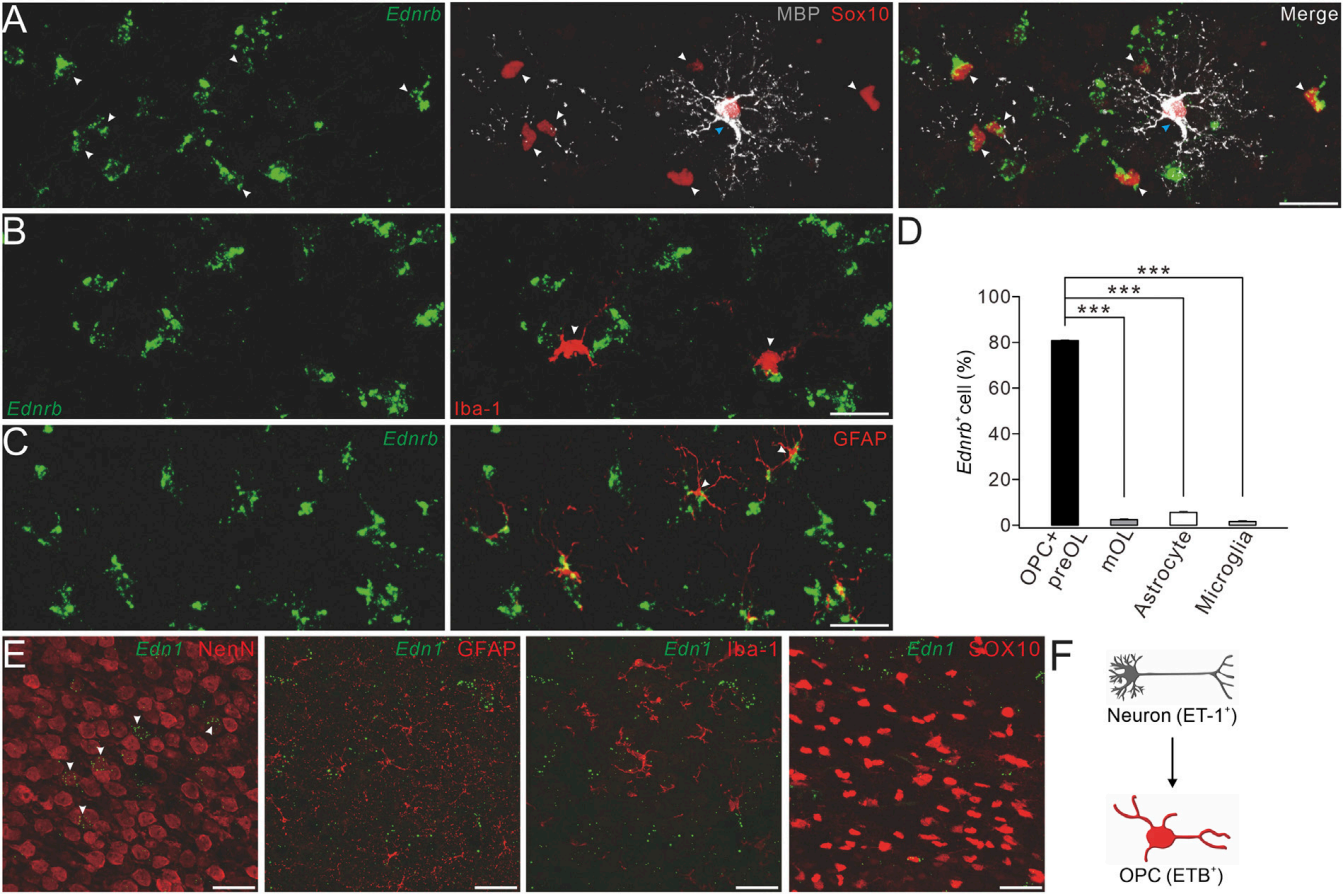
Expression of ETB and ET-1 in OPCs and neurons. **(A)** Representative control cerebral cortex section (P4) with *Ednrb* RNAScope probe and double-immunostaining of MBP and SOX10. White arrowheads, *Ednrb* ^+^MBP^−^SOX10^+^ OPCs; and blue arrowheads, *Ednrb* ^−^MBP^+^SOX10^+^ mature OLs (scale bar, 50 μm). **(B)** *Ednrb* RNAScope and immunostaining of Iba-1 in the control cerebral cortex (P4). Arrowheads, *Ednrb* ^−^Iba-1^+^ microglia (scale bar, 50 μm). **(C)** *Ednrb* RNAScope and immunostaining of GFAP in control cerebral cortex (P4). Note that the arrowheads show *Ednrb* ^−^GFAP^+^ astroglia (scale bar, 50 μm). **(D)** Percentages of *Ednrb* ^+^ cells among OPCs and pre-OLs (80.8% ± 0.4%) and mature OLs (2.4% ± 0.3%; *p* < 0.001 vs*.* OPCs and pre-OLs), astrocytes (5.6% ± 0.3%; *p* < 0.001 vs*.* OPCs and pre-OLs), and microglia (1.6% ± 0.3%; *p* < 0.001 vs*.* OPCs and pre-OLs). **(E)** Representative *Et-1* RNAScope and immunostaining of NeuN, GFAP, Iba-1, or SOX10 in the control cerebral cortex (P4). Arrowheads, *Et-1* ^+^NeuN^+^ neurons (scale bars, 50 μm). **(F)** Schematic of the ET-1/ETB signaling module.

First, we applied RNAscope *in situ* hybridization, in combination with protein immunostaining, to examine the expressions of *Ednrb* and *Edn1*, which encode ET-1 in major cell types in the brain. We detected *Ednrb* in SOX10^+^MBP^−^ cells, but not in SOX10^+^MBP^+^ cells, indicating that *Ednrb* is solely expressed in OPCs and pre-OLs (Figure 3A). We then investigated whether Ednrb is expressed in microglia and astroglia and found that it did not colocalize with Iba-1, a putative marker for microglia (Figure 3B), but colocalized with GFAP, a putative marker for astroglia, at a very low fraction (Figure 3C). The percentages of *Ednrb* ^+^ cells in premylienating OLs (OPCs + pre-OLs), mature OLs, microglia, and astroglia are presented in Figure 3D. Despite the rather weak detection in astroglia, it was enough to conclude that *Ednrb* is mainly and robustly expressed in premylienating OLs.

We next determined the location of *EDN1* expression using RNAscope and immunostaining with antibodies to NeuN, Iba-1, GFAP, and SOX10. Our results showed that the *EDN1* signal overlapped well with NeuN, but not with Iba-1, GFAP, or SOX10 (Figure 3E), suggesting that ET1 is mainly expressed in neurons. Taken together, we found that ET-1 is expressed in neurons and ETB in OPCs, suggesting that ET-1 and ETB work together to affect neuron–OPC interactions (Figure 3F).

### ETB ablation impedes OPC differentiation and impairs CNS myelination

**FIGURE 4:**
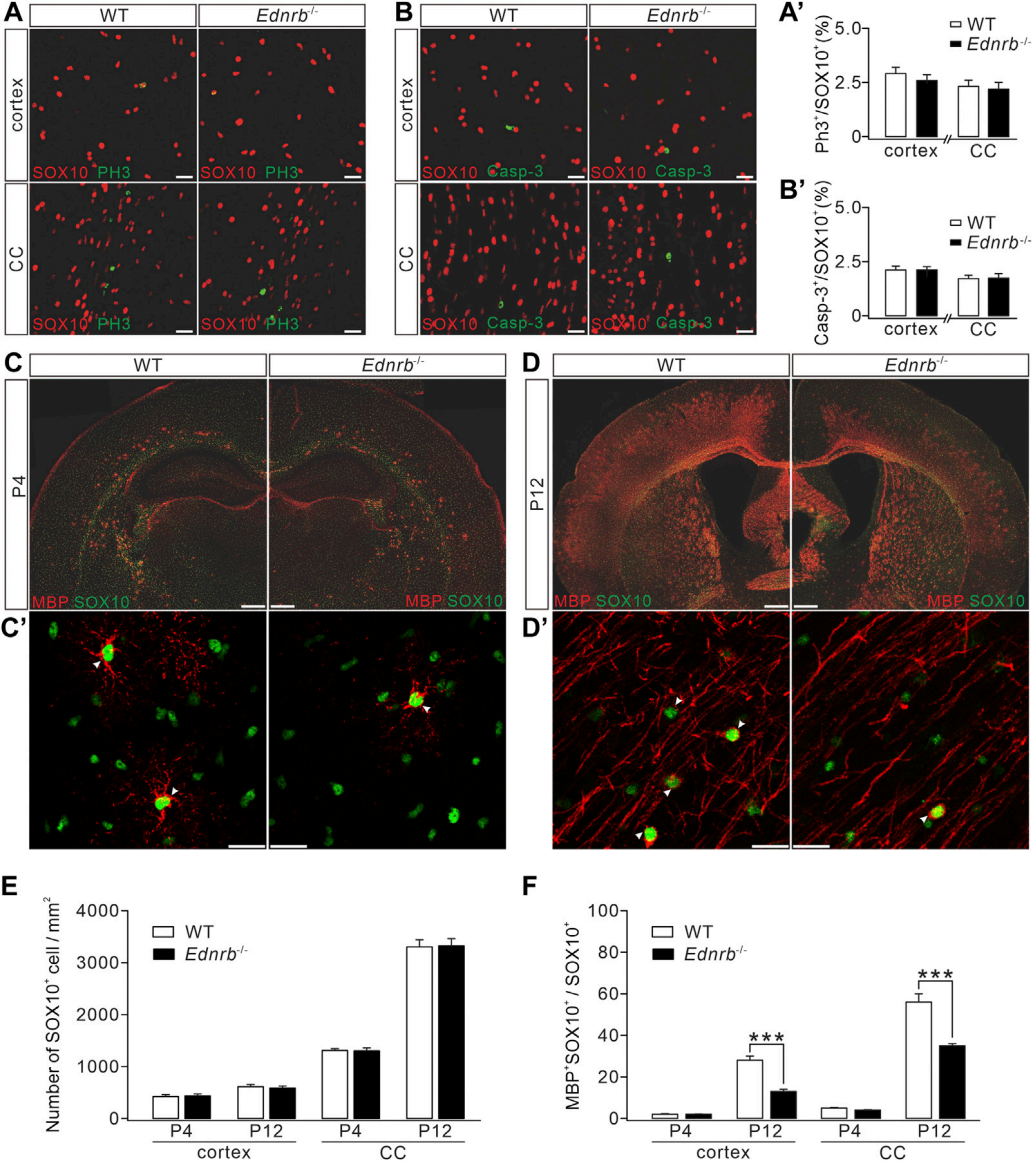
Effects of *Ednrb* ablation on MBP expression. **(A)** Double-immunostaining of SOX10 and PH3 in the cerebral cortex and corpus callosum (CC) of WT and *Endrb* ^*−/−*^ mice (P12). Scale bars: 50 μm. **(A** *′* **)** Percentages of PH3^+^ cells among SOX10^+^ cells: 2.8% ± 0.2% (WT; *n* = 5) and 2.5% ± 0.4% (*Endrb* ^*−/−*^; *n* = 5; *p* = 0.32) in the cortex; 2.3 %± 0.2% (WT; *n* = 5) and 2.1% ± 0.3% (*Endrb* ^*−/−*^; *n* = 5; *p* = 0.87) in the corpus callosum. **(B)** Double-immunostaining of SOX10 and caspase-3 (Casp-3) in the cerebral cortex and corpus callosum of WT and *Endrb* ^*−/−*^ mice (P12). Scale bars: 50 μm. **(B′)** Percentages of caspase-3^+^ cells among SOX10^+^ cells: 2.1% ± 0.2% (WT; *n* = 3) and 2.1% ± 0.2% (*Endrb* ^*−/−*^; *n* = 3; *p* = 0.93) in the cortex; 1.7% ± 0.2% (WT; *n* = 3) and 1.7% ± 0.2% (*Endrb* ^*−/−*^; *n* = 3;*p* = 0.98) in the corpus callosum. **(C)** MBP and SOX10 in P4 mouse brain. Scale bars: 1 mm. (**C′**) Magnified view (cerebral cortex) showing the colocalization of SO X10 and MBP. Scale bars: 50 μm. **(D)** MBP and SOX10 in P12 mouse brain. Scale bars: 1 mm. (**D′**) Magnified view (cerebral cortex) showing the colocalization of SOX10 and MBP. Scale bars: 50 μm. **(E)** Density of SOX10^+^ cells. In the cortex: 419 ± 37/mm^2^ (WT + P4; *n* = 5) and 432 ± 37/mm^2^ (*Endrb* ^−/−^ + P4; *n* = 5; *p* = 0.79); 607 ± 42/mm^2^ (WT + P12; *n* = 5) and 581 ± 38/mm^2^ (*Endrb* ^−/−^ + P12; *n* = 5; *p* = 0.62). In the corpus callosum: 1,289 ± 34/mm^2^ (WT + P4; *n* = 5) and 1,290 ± 58/mm^2^ (*Endrb* ^−/−^ + P4; *n* = 5; *p* = 0.13); 3,271 ± 137/mm^2^ (WT + P12; *n* = 5) and 3,290 ± 138/mm^2^ (*Endrb* ^−/−^ + P12; *n* = 5; *p* = 0.74). **(F)** Percentages of MBP^+^SOX10^+^ cells in SOX10^+^ cells. In the cortex: 2% ± 0.4% (WT + P4; *n* = 5) and 2% ± 0.2% (*Endrb* ^−/−^ + P4; *n* = 5; *p* = 0.61); 28% ± 2% (WT + P12; *n* = 5) and 13% ± 1% (*Endrb* ^−/−^ + P12; *n* = 5; *p* < 0.001). In the corpus callosum: 5% ± 0.4% (WT + P4; *n* = 5) and 4% ± 0.4% (*Endrb* ^−/−^ + P4; *n* = 5; *p* = 0.06); 56% ± 4% (WT + P12; *n* = 5) and 35% ± 1% (*Endrb* ^−/−^ + P12; *n* = 5; *p* = 0.0005). ***p < 0.001.

Having demonstrated that ETB is expressed in premylienating OLs, a critical question was whether ETB deficiency causes hypomyelination. To address this question, we compared OL development in wild-type (*WT*) and *Ednrb* ^−/−^ mice. First, the proliferation of OPCs followed by deletion of *Ednrb* was investigated using immunohistochemical staining with the antibodies against PH3 and SOX10. Our results showed that the density of SOX10^+^ cells was not affected by *Ednrb* ablation in the cerebral cortex and corpus callosum (Figure 4A). Moreover, the percentage of proliferating SOX10^+^PH3^+^ OPCs among SOX10^+^ OLs was not altered by *Ednrb* ablation in *Ednrb* ^−/−^ mice (Figures 4A, A’). Next, a caspase-3 assay was used to determine whether *Ednrb* ^−/−^ ablation causes apoptosis, and it showed that the percentage of caspase-3^+^SOX10^+^ OLs among all SOX10^+^ OLs in *Ednrb* ^−/−^ mice was comparable to that in *WT* mice (Figures 4B,B’). Therefore, neither the proliferation nor the survival of OPCs was affected by ETB deletion.

We next examined myelin formation in *Ednrb* ^−/−^ mice. Immunohistochemical staining showed that MBP expression was lower in the cerebral cortex and corpus callosum of *Ednrb* ^−/−^ mice than in *WT* mice at P4 (Figure 4C), suggesting a hypomyelination phenotype in *Ednrb* ^−/−^ mice at an early postnatal age. To determine whether this phenotype persists with development, we applied MBP staining to *WT* and *Ednrb* ^−/−^ mice at P12 and found that the difference in MBP expression became greater, showing that there were fewer MBP^+^ cells in the cerebral cortex and the corpus callosum of *Ednrb* ^−/−^ mice (Figure 4D). Counting the number of SOX10^+^ OLs in mice at P4 and P12 showed that SOX10^+^ OLs were unaffected by the deletion of *Ednrb* (Figure 4E). In contrast, the percentage of mature OLs (MBP^+^SOX10^+^) among OLs was always lower in *Ednrb* ^−/−^ mice than in *WT* mice at P4 and P12 (Figure 4F). Taken together, the immunohistochemical assays led to the conclusion that *Ednrb* deletion leads to a myelin defect.

### ETB deficiency exaggerates the WMI-induced impairment of OL maturation

**FIGURE 5:**
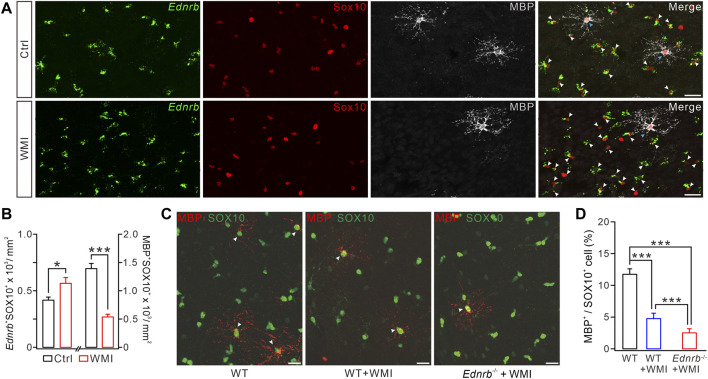
ETB deficiency exaggerates WMI-induced MBP reduction. **(A)** *Ednrb* RNAScope and immunostaining of MBP and SOX10 in control and WMI cerebral cortex (P10) (white arrowheads, *Ednrb* ^+^ OPCs; blue arrowheads. Mature OLs; Scale bars, 50 μm). **(B)** Density of *Ednrb* ^+^SOX10^+^ cells: 417 ± 27/mm^2^ (control; *n* = 4) and 566 ± 51/mm^2^ (WMI; *n* = 4; *p* = 0.023). Density of MBP^+^SOX10^+^ cells: 1,393 ± 90/mm^2^ (control; *n* = 4) and 537 ± 48/mm^2^ (WMI; *n* = 4; *p* < 0.001). **(C)** Double-immunostaining of MBP and SOX10 in WT, WT + WMI, and *Endrb* ^*−/−*^ + WMI cerebral cortex (P10) (white arrowheads. MBP^+^ mature OLs; scale bars, 50 μm). **(D)** Percentages of MBP^+^ cells among SOX10^+^ cells: 11.7% ± 0.8% (WT; *n* = 5), 4.8% ± 0.9% (WT + WMI; *n* = 5; *p* = 0.00019 vs*.* WT), and 2.6% ± 0.6% (*Endrb* ^*−/−*^ + WMI; *n* = 5; *p <* 0.001 vs*.* WT; *p* < 0.001 vs*.* WT + WMI). *p < 0.05. ***p < 0.001.

We next investigated the effect of ETB ablation on the WMI-induced myelin deficit. To this end, we used an *Ednrb* RNAscope probe in combination with SOX10 and MBP immunostaining to quantify the numbers of *Ednrb* ^+^ OPCs and pre-OLs in the control and WMI mice. Our results showed that the number of *Ednrb* ^+^SOX10^+^MBP^−^ cells increased by ∼30% in the cerebral cortex following WMI (Figures 5A, B). Meanwhile, the number of *Ednrb* ^−^MBP^+^SOX10^+^ cells was reduced by ∼70% (Figures 5A, B). These results strengthened our conclusion from the scRNA-Seq data (Figure 2G), demonstrating that WMI arrests OLs at the premylienating stage.

Since both WMI and ETB deficiency cause a myelin deficit by inhibiting OPC differentiation, it was possible that ETB deficiency may exaggerate the defective myelin sheath caused by WMI. To address this question, we assigned the following three groups: *WT* mice, *WT* mice with WMI surgery, and *Ednrb* ^*−/−*^ mice with WMI surgery. In each of these groups, we applied double immunostaining for MBP and SOX10 to the cerebral cortex. We found that the density of SOX10^+^MBP^+^ cells was significantly decreased in *WT* mice after WMI surgery, compared to control *WT* mice (Figures 5C, D), and this reduction was more severe in the condition of ETB deletion (Figures 5C, D). Therefore, ETB deficiency exaggerates the WMI-induced impairment of OL maturation.

### ET-1 ameliorates the defective OL maturation induced by oxygen-glucose deprivation (OGD)

**FIGURE 6:**
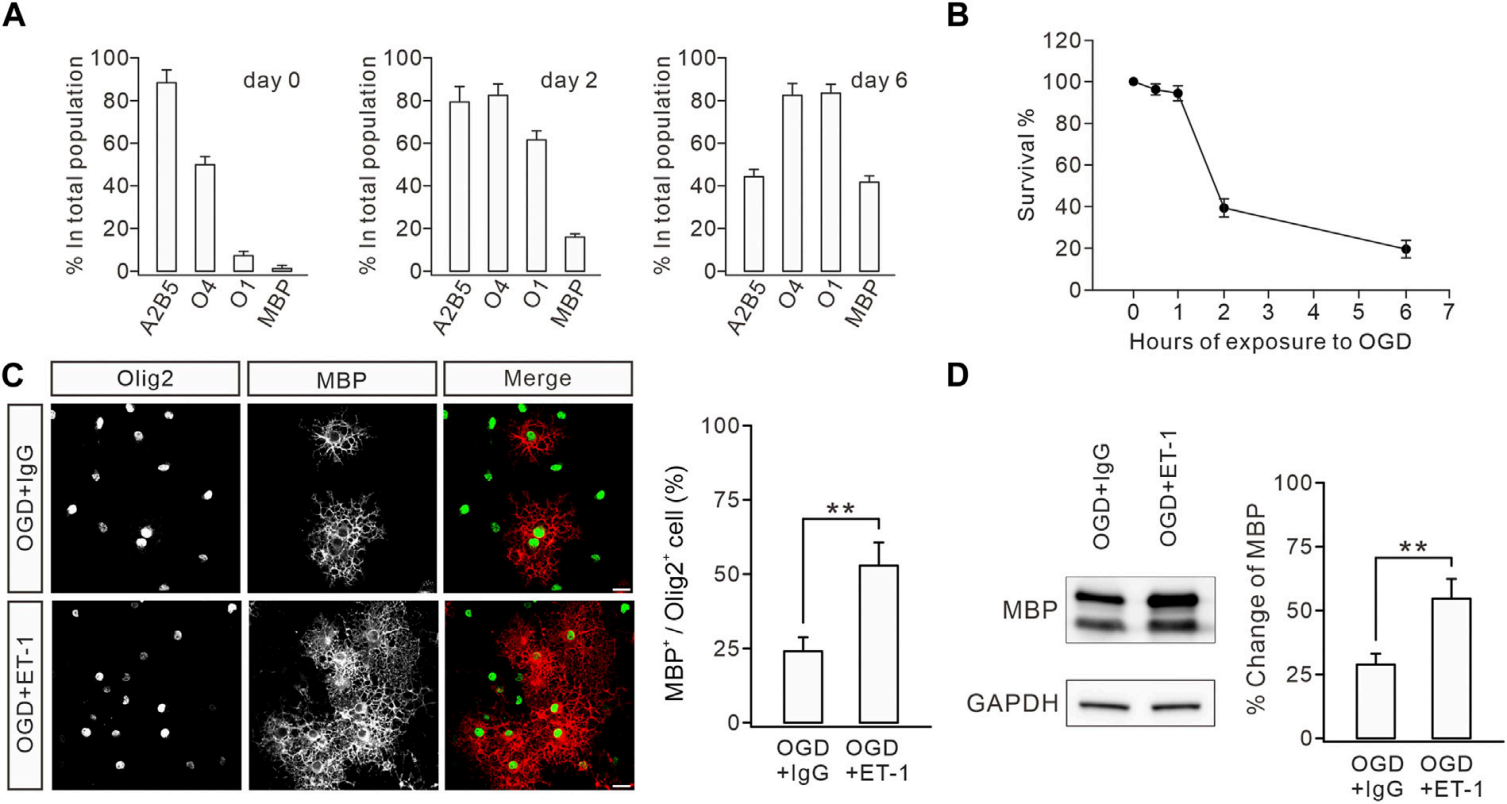
ET-1 ameliorates the OGD-induced defect in OL maturation. **(A)** Stage-specific cultures. Cultures were allowed to differentiate for 0, 2, or 6 days and stained for the OL stage-specific markers A2B5, O4, O1, and MBP. Bar graph shows the percentages of immunolabeled cells in the total population. For day 0: 88.2% ± 6.1% (A2B5), 49.8% ± 3.8% (O4), 7.0% ± 2.0% (O1), and 1.8% ± 0.8% (MBP); *n* = 5. For day 2: 79.2% ± 7.2% (A2B5), 82.4% ± 5.3% (O4), 61.4% ± 4.2% (O1), and 16.0% ± 1.5% (MBP); *n* = 5. For day 6: 44.2% ± 3.4% (A2B5), 82.4% ± 5.6% (O4), 83.4% ± 4.2% (O1), and 41.6% ± 3.0% (MBP); *n* = 5. **(B)** Effect of OGD duration on OPC death. Cultures were exposed to OGD for 0, 0.5, 1, 2, or 6 h, and cell survival was determined at 24 h. 0.5 h, 96.2% ± 2.7%, *n* = 5; 1 h, 94.6% ± 3.6%, *n* = 5; 2 h, 39.6% ± 4.3%, *n* = 5; 6 h, 19.8% ± 4.3%, *n* = 5. **(C)** After OGD, cultured OPCs were treated with IgG or ET-1, as well as T3, and immunostained with Olig2 and MBP antibodies (scale bars, 20 μm). The percentages of MBP^+^ cells in Olig2^+^ cells: 23.8% ± 4.2% (IgG; *n* = 5) and 52.8% ± 7.7% (Et-1; *n* = 5); *p* = 0.006. **(D)** Representative blots and percentage changes of MBP expression in cultured OLs with IgG or ET-1. MBP expression is normalized to the corresponding GAPDH. IgG, 28.8% ± 4.2% (*n* = 5); ET-1, 54.6% ± 7.6% (*n* = 5; *p* = 0.011). Gray dots indicate individual data points. **p < 0.01.

The impaired myelination caused by ETB deficiency prompted us to determine whether functional enhancement of ETB promotes myelin formation. We tested this hypothesis in OPC cultures derived from embryonic rats (Zhou et al., 2018; Wang et al., 2022), in which OPCs were exposed to OGD to mimic WMI conditions since cerebral WMIs, such as PVL, are caused by hypoxic–ischemic insults in preterm infants (Baldassarro et al., 2020). We optimized the durations of OGD exposure to assess its toxicity in OPC cultures. The time course of the vulnerability of OPCs showed that 1 h of OGD exposure only affected a minority of cells, whereas 2 h of OGD exposure eliminated >50% of cells by 24 h (Figure 6A), consistent with previous reports (Deng et al., 2003). Because we had demonstrated that WMI surgery did not change the survival rate of OLs (Figure 1F), 1 h of OGD exposure was chosen to mimic the WMI surgery. OPCs were exposed to OGD only once on day 1, followed by incubation with IgG (100 μM) or ET-1 (100 μM) for two days. The cultured OLs were stained with MBP and Olig2 antibodies to calculate the percentages of mature OLs. Our results showed that ET-1 significantly increased the percentage of MBP^+^ cells among Olig2^+^ cells by 31% (Figure 6B). Furthermore, Western blots showed that ET-1 yielded a remarkable increase in the expression of MBP, compared to the control IgG (Figure 6C). These results suggested that stimulating ET-1-ETB signaling can rescue the impaired OL maturation induced by preterm WMI.

## Discussion

Using scRNAseq analysis, we revealed that ETB is specifically expressed in premylienating OLs and that preterm WMI, which impedes OPC differentiation, leads to an increase of ETB^+^ OPCs and pre-OLs. Moreover, we demonstrated that neuron-derived ET-1 and OPC-derived ETB promote the differentiation of OPCs and the maturation of OLs. In summary, our research suggests a role of ET-1/ETB signaling in OPC differentiation and WMI and provides new insight into the pathological mechanisms underlying preterm WMI and OPC transplantation therapy for this disease.

### The ET-1/ETB complex for OPC differentiation

Oligodendrogenesis is composed of three stages: OPCs, pre-OLs, and mature OLs, which eventually wrap around axons and produce myelin sheath (Baumann and Pham-Dinh, 2001; Elbaz and Popko, 2019). In addition to intrinsic factors that are mostly transcription regulators, extrinsic factors, such as secreted proteins (Moore et al., 2011), adhesion molecules (Popko, 2003), and extracellular matrix (Marangon et al., 2020), also regulate OPC differentiation and myelination by interacting with corresponding partners on OPCs. For example, PSA-NCAM inhibits myelination (Charles et al., 2002) and Jagged-1 binds to Notch receptors in OPCs to inhibit OPC differentiation (Jurynczyk et al., 2008), whereas Neuregulin-1 promotes OPC differentiation by binding to ErbB (Navel and Salzer, 2006). Overall, neuron-derived factors that act on OL development are not yet well-understood.

ET-1/ETB signaling is present as a complex in tumor cells and the vascular system and participates in regulating the blood supply (Kostov, 2021). Our research indicates that the ET-1/ETB complex is responsible for the interaction between neurons and OLs, providing new evidence for the role of this complex in the CNS. We showed that ETB was more specifically expressed in OPCs and pre-OLs, suggesting that ETB is a marker of premylienating OLs. We also found that knocking down ETB decreased mature OLs, while activating ET-1/ETB signaling promoted OL maturation, indicating that the ET-1/ETB complex is a positive regulator of OPC differentiation. It was observed that ET-1 mainly existed in neurons, which seems inconsistent with a previous work showing that ET-1 is also released from astroglia (Gadea et al., 2009). However, the expression and release of ET-1 were only examined in the subventricular zone in that study (Gadea et al., 2009). Therefore, their study did not exclude the source of ET-1 in mature neurons, a scenario investigated in the present work.

It has been shown that the bi-directional interactions between neurons and OLs are multistage and multiform. For example, early-stage OPCs form synaptic communication with neurons (Lin and Bergles, 2004; Moura et al., 2022), suggesting that neuronal activity acts on OLs (Zonouzi et al., 2011; Nagy et al., 2017). In the mature CNS, optogenetic stimulation that enhances neuronal activity increases OPC differentiation and the number of mature OLs (Gibson et al., 2014). In addition, motor learning increases or decreases the occurrence of OLs in the motor cortex (Bacmeister et al., 2020), and the activation of axons in the corpus callosum increases oligodendrogenesis and myelination in fear behavior (Mitew et al., 2018). Considering that the ET-1/ETB complex is a positive regulator of neuron–OPC interactions, it will be interesting to investigate whether this complex is involved in regulating myelin-related behaviors. In addition, some of the conclusions of the present work were based on global knockout and cell culture experiments; thus, cell-specific knockout of ET-1 and ETB would be required to better clarify the roles of ET-1/ETB signaling in myelin development and WMI.

### A hypoxic–ischemia model in newborn mice mimics preterm WMI in the human embryo

During 23–32 weeks of gestation, the OL lineages are mainly OPCs and pre-OLs (van Tilborg et al., 2018a). This period is not only a key stage for OL maturation but is also a high-risk time for preterm WMI. Various factors such as embryonic malnutrition or hypoxia–ischemia can lead to the interruption of OPC differentiation and myelin sheath formation and result in white matter damage and permanent myelin sheath dysplasia (Ma and Zhang, 2015). Development of OLs from P1 to P7 in rodents is comparable to that of 23–36 weeks of gestation in humans (Hagberg et al., 2002; Craig et al., 2003). Therefore, we applied hypoxia–ischemia to neonatal mice (at P3) to recapitulate the characteristic changes due to preterm WMI. Indeed, we found that the development of myelin sheaths was inhibited in mice exposed to hypoxia–ischemia. However, unlike the extensive death of pre-OLs in infants with moderate or severe WMI (Back and Rosenberg, 2014), hypoxia–ischemia did not induce an excessive loss of OPCs or pre-OLs, as indicated by stable numbers of caspase-3^+^ and SOX10^+^ cells. Caspase-3 activation is tightly associated with the severity of cell death (Hisahara et al., 2003), based on the finding that pre-OLs degenerate due to caspase-3 activation in perinatal rodents exposed to moderate hypoxia–ischemia (Calvert and Zhang, 2005; Pedroza-Garcia et al., 2022). In support of our conclusions, it has been reported that the number of pre-OLs increases 2–3-fold within 24 h after hypoxia–ischemia in neonatal rats (Wahl and Schwab, 2014) and that of PDGFRα^+^ OPCs gradually increases with hypoxia–ischemia (Miyamoto et al., 2015), indicating that OPCs and pre-OLs develop normally at the early stage of hypoxia–ischemia.

We established a relatively mild WMI model, in which OPCs and pre-OLs were not subject to the threat of death. This model also matches an *in vitro* OGD model, in which cultured OLs are briefly deprived of glucose and oxygen. We believe that this less lethal model allows screening for signaling molecules affected in the early stage after WMI, thus providing a theoretical basis for early intervention in WMI. Indeed, our results reveal a series of molecules related to various cellular processes during WMI. Interestingly, we show, for the first time, that ETB, a receptor mediating neuron–OPC interaction, is sensitive to early WMI.

### Neuron–OPC interaction in WMI

WMI causes severe damage to the CNS development of premature infants, and there is no effective treatment for neonatal WMI (van Tilborg et al., 2018b). The microenvironment after WMI is harmful to OPC differentiation, providing a therapeutic target to cure preterm WMI. It has been demonstrated that astrocytes, microglia, vascular endothelial cells, and extracellular matrix components dominate an unfavorable microenvironment for the negative regulation of OPC development during WMI (Riddle et al., 2011; Kurachi et al., 2016; de Jong et al., 2020; Traiffort et al., 2020; Motavaf and Piao, 2021). Moreover, the unfavorable microenvironment also affects the development of implanted OPCs in cell transplantation therapy: exogenous OPCs can migrate to the demyelinating lesions, but fail to differentiate into mature OLs (van Tilborg et al., 2016). Therefore, improving the white matter microenvironment is beneficial to the development of transplanted OPCs. For example, the interaction between microvascular endothelial cells and OLs is the key to OL development in WMI (Hamanaka et al., 2018b) and is conducive to white matter remodeling (Zacchigna et al., 2008). Endothelial cells secrete a variety of nutrients to mediate the signal coupling between blood vessels and OLs and to support the survival and proliferation of OPCs (Hamanaka et al., 2018a). Such nutrient molecules include endothelial-derived growth factor, brain-derived neurotrophic factor, fibroblast growth factor-2, transforming growth factor-*β*, and vascular endothelial growth factor-A (Asakura et al., 1997; Zhou et al., 2006; Gonzalez-Perez and Alvarez-Buylla, 2011; Ramos-Cejudo et al., 2015). However, it should be noted that a single signaling pathway or a single cell type may not be enough to rescue the damage caused by preterm WMI. Since neuron–OPC interaction plays a crucial role in synaptic activity, its roles in the WMI also need to be addressed. Our scRNA-seq investigation demonstrated that, besides ETB, a considerable number of other neuron-derived molecules were affected by WMI. Therefore, studying neuron–OPC interactions during WMI may provide new therapeutic targets aimed at preterm WMI.

## Conclusion

Using single-cell RNA sequencing, we revealed that ET-1 and ETB are a pair of signaling molecules responsible for the interaction between neurons and OPCs and that preterm WMI led to an increase in the number of ETB-positive OPCs and premyelinating OLs.

## Data Availability

The datasets presented in this study can be found in online repositories. The names of the repository/repositories and accession number(s) can be found below: NCBI GEO: GSE225580.
