# Supplementary figures and images for: Endothelin-1–Endothelin receptor B complex contributes to oligodendrocyte differentiation and myelin deficits during preterm white matter injury
Source: Front Cell Dev Biol. 2023 Mar 17;11:1163400. doi: 10.3389/fcell.2023.1163400 (PMC10063893; doi:10.3389/fcell.2023.1163400)

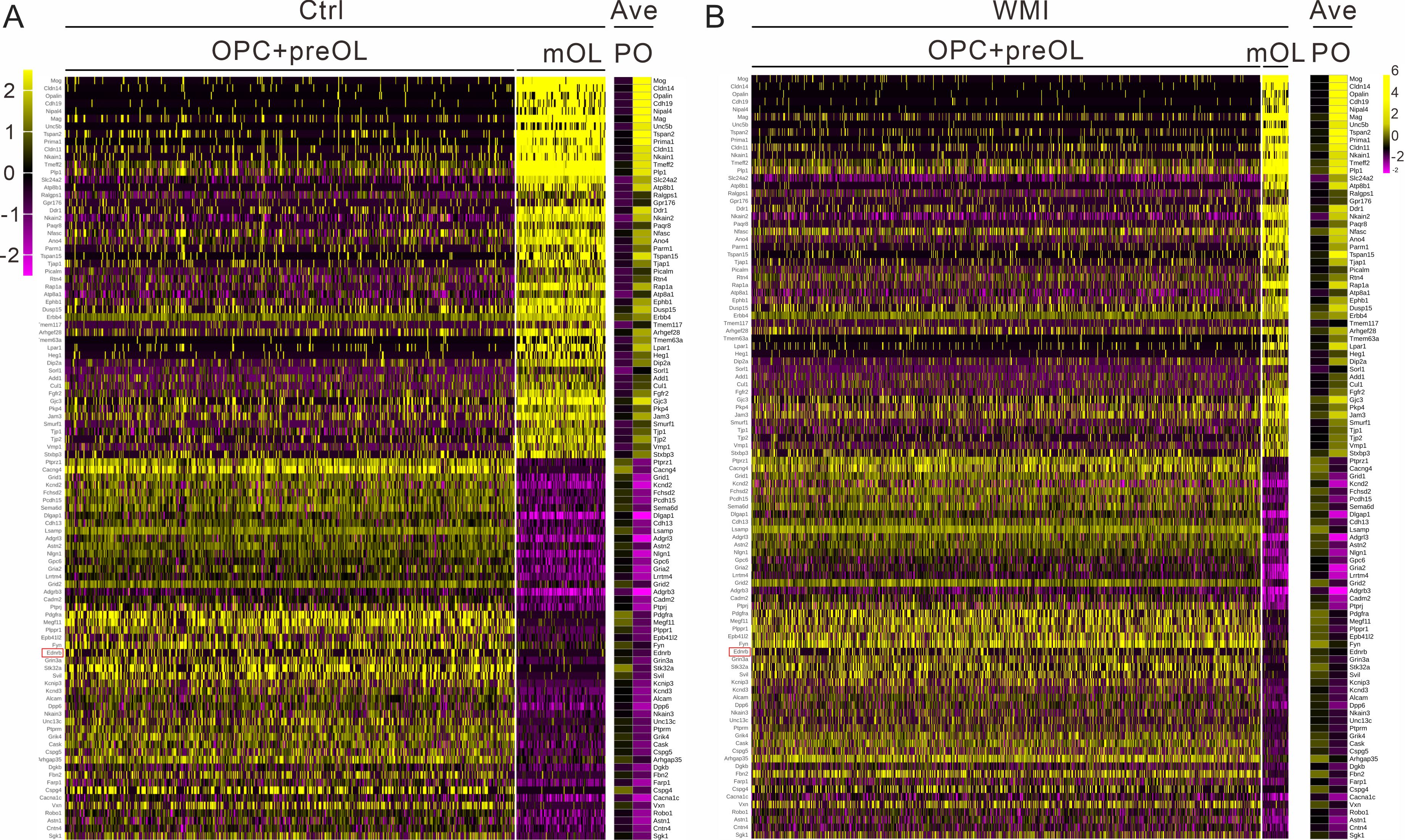

Supplement: Supplementary file 1 [file Image1.JPEG]
